# Pilot Study: Soluble LPS/IgG Milk Complexes in Relationship to Early Lactation Acute Mastitis in Dairy Cows

**DOI:** 10.3390/ani16020310

**Published:** 2026-01-20

**Authors:** Suzanne M. Hurst, Richard Laven, Anton Pernthaner

**Affiliations:** 1 Koru Biotech Solutions Ltd., Estendart Research Centre, Aviation Way, Massey University Campus, Palmerston North 4472, New Zealand; tony@korubiotech.com; 2 School of Veterinary Science, Massey University, University Avenue, Fitzherbert, Palmerston North 4474, New Zealand; r.laven@massey.ac.nz

**Keywords:** LPS, acute mastitis, soluble immune complexes, LPS/IgG, Gram-negative bacteria, dairy, lactation, intramammary

## Abstract

Prevalence of LPS in Gram-negative bacterial udder infections determines mastitis severity and disease prognosis. This pilot study explores the notion that milk-soluble (s) LPS/IgG complex levels in dairy cows link mastitis severity to intramammary Gram-negative infections during early lactation. Milk, within a single herd, was analysed from (i) 34 early lactating cows with acute mastitis and (ii) milk selected from peak lactation cows displaying either healthy (SCC < 100 × 10^3^ cells/mL, *n* = 146) or subclinical mastitis (SCC > 150 × 10^3^ cells/mL, *n* = 135) characteristics. Milk was assessed for (i) sLPS/IgG using an “in-house” ELISA, (ii) udder inflammation using LDH activity, and (iii) bacterial presence applying on-farm and standard microbiological laboratory techniques. Mean milk sLPS/IgG absorbances in acute mastitis cows were higher than those detected in healthy and subclinical mastitis cows, with mean differences of 0.35 (95% CI, 0.28 to 0.42) and 0.36 (95% CI, 0.28 to 0.44), respectively. On day 1 of acute mastitis, sLPS/IgG levels in milk containing only Gram-positive bacteria ranged from OD 0.04 to 0.14 (median = 0.1). In contrast, sLPS/IgG levels ranging from OD 0.27 to 1.42 (median = 0.58) and from 0.02 to 1.67 (median = 0.21) were detected in milk containing only Gram-negative bacteria or both Gram-negative and Gram-positive bacteria (i.e., polymicrobial), respectively. Furthermore, differential milk sLPS/IgG absorbance profiles (observed during the testing period days 1–3) were observed in cows with acute mastitis caused by Gram-positive, Gram-negative or polymicrobial infections. Our preliminary findings support the notion that milk sLPS/IgG complexes provide a link between mastitis severity and intramammary Gram-negative infections in dairy cows during early lactation.

## Simple Summary

Acute udder inflammation (mastitis) often occurs in cows early in the lactation cycle. Mastitis is primarily caused by a wide range of bacteria including both Gram-negative and Gram-positive species. Gram-negative infections frequently result in severe mastitis due to the presence of pro-inflammatory compounds known as lipopolysaccharides (LPS). During the immune response, removal of LPS involves antibodies in the udder that form immune complexes, which support the clearance of bacteria and toxins from the udder and help the cow return to good health and milk production. This study identifies soluble LPS/IgG immune complexes in milk and explores the notion that milk-soluble (s) LPS/IgG complex levels in dairy cows link mastitis severity to intramammary Gram-negative infections during early lactation. We measured soluble milk LPS/IgG complexes alongside a known marker of udder inflammation (lactate dehydrogenase activity) in healthy cows and cows with subclinical or acute mastitis. Our results showed that increased milk levels of soluble LPS/IgG complexes were associated with acute udder inflammation caused by Gram-negative bacterial infections. This suggests measuring milk-soluble LPS/IgG complexes could be useful in the detection and management of acute mastitis in dairy cows.

## 1. Introduction

The mammary gland is under constant exposure to opportunistic environmental bacterial pathogens [1]. In a healthy lactating cow, intramammary immune responses are highly coordinated to minimise infection and mucosal tissue inflammation and maintain optimal milk production [2]. However, nutritional and metabolic demands after calving together with postpartum immune deficiencies increase the risk of bacterial infections resulting in acute mastitis during early lactation [3]. Acute mastitis is typically identified by visible clinical signs of inflammation of the udder as well as changes to milk quality [4]. Identification of the causative organism in a case of acute mastitis can identify optimal treatment and provide information that is valuable for prevention of further cases. Microbiological culture of milk is the standard laboratory approach to identifying the cause of mastitis but normally requires 24–48 h to obtain a diagnosis and frequently fails to identify the causative pathogen because of “no growth”, contamination or polymicrobial identification [5]. Furthermore, on their own, microbiology-based approaches lack the capability to link bacterial identification with inflammation and the severity of acute mastitis. Whilst Gram-positive infections (e.g., *Streptococci* or *Staphylococci*) can cause acute mastitis in early lactation cows, Gram-negative bacterial infections, especially environmental pathogens (e.g., *Escherichia coli* [*E. coli*]), may cause severe intramammary inflammation that results in significant loss of milk production and even loss or removal of the cow from the herd [6,7]. This is a major concern for dairy farmers, as Gram-negative infections are the largest contributors to the negative economic impact that acute mastitis has on the global dairy industry [8].

Lipopolysaccharide (LPS) compounds located on the surface of Gram-negative bacteria can have a profound effect on the mammalian immune system [9]. In mastitis, it is these LPS compounds which are the principal drivers of the clinical signs seen in cows with Gram-negative mastitis [10,11], with instillations of LPS (20–100 µg/mL) into the mammary gland shown to evoke acute inflammation and mucosal tissue damage within 6 h of administration. Current methods for direct measurements of milk LPS levels during mastitis target its endotoxin activity using the enzymatic activity of *Limulus amebocyte* lysate (LAL) [12,13], but despite assay standardisation [13], the detection of LPS endotoxicity does not necessarily reflect its pro-inflammatory potential. This is primarily due to the presence of compounds in the milk of healthy lactating cows, such as casein and lactoferrin [14], that sequester the endotoxic and/or pro-inflammatory potential of any LPS present. However, acute mastitis causes a rapid proteolysis of these key LPS-sequestering milk proteins [15] that results in the dissociation of LPS which, even in relatively small amounts (~10^−9^ M), will augment inflammation. Therefore, identifying milk biomarkers that link LPS to intramammary inflammatory status would provide a better insight into the involvement of Gram-negative infections in early lactation mastitis.

Whilst this research area is still in its infancy, studies evaluating events downstream from Gram-negative bacterial LPS cellular responses have identified a number of potential metabolic and inflammatory mediators [11,16]; however, the identification of milk biomarkers that specifically link Gram-negative infections to acute mastitis status remain elusive. The interplay between intramammary immunoglobulins (Ig) during a bacterial infection is integral to an effective mucosal defence system [17]. This is especially important in bovine udder infections involving environmental Gram-negative coliforms, where the transfer of antigen-specific IgG from the circulation into the mammary gland correlates with mastitis severity [6,7]. Bacterial neutralisation and eventual clearance involve the formation of antigen-Ig complexes within the udder to coordinate appropriate immune responses. It is therefore feasible that the local formation of LPS/IgG complexes within the mammary gland during Gram-negative bacterial infections could signify the presence of LPS/endotoxin-driven mastitis and link Gram-negative bacteria infections to intramammary inflammatory status.

The biological properties of immune complexes are diverse and dependent upon the “molar ratio of antigen and antibody, size, charge, antibody affinity, and antibody subclass” [18]. Soluble immune complexes, however, found in blood during bacterial or viral infections are associated with pro-inflammatory events and promotion of chronic tissue inflammation and poor disease prognosis [18,19]. In a previous study, we showed that the elevation of preformed soluble LPS/IgG complexes in milk correlated with postpartum acute intramammary inflammation caused by *E. coli* infections [20]. It is currently unknown whether soluble antigen-specific immune complexes, such as LPS/IgG, formed locally within the udder during a Gram-negative bacterial infection, could imply mastitis severity.

In this pilot observational study, we explore the notion that milk soluble (s) LPS/IgG complex levels in dairy cows link intramammary Gram-negative infections to mastitis severity in early lactation.

## 2. Materials and Methods

### 2.1. Animals

#### 2.1.1. Ethical Approval

Approval for this pilot study was obtained from Animals Ethics Committee (AEC), Massey University/Te Kunenga ki Pūrehuroa, Palmerston North, NZ. File number, AEC 22/41 and approved 19/08/2022.

#### 2.1.2. Acute Mastitis Identification and Milk Sampling/Processing

A commercial, predominantly pasture-fed and pasture-based ~1100 cow dairy herd in the southern part of the North Island of New Zealand (NZ) was used for the study. The herd was spring calving (mid-July to mid-September 2022) and consisted of Holstein-Friesian and Holstein-Friesian-Jersey cross cows.

We received quarter milk samples from cows during early lactation over a period of 8 weeks. Experienced farm technicians, during routine milking, observed cows for signs of acute mastitis, i.e., swelling, redness, pain and heat of the mammary gland and a positive California Mastitis Test (CMT), following routine on-farm protocols. All cows diagnosed with acute mastitis were immediately treated with injectable NSAIDs and, in addition to milking, milk from the affected quarter was stripped every ~12 h. If Gram-positive bacteria were identified (by on-farm Mastatest analysis [12–24 h later]) in the aseptic milk samples, cows also received appropriate intramammary antibiotics as prescribed by the farm veterinarian. Cows identified as having only Gram-negative bacteria in their milk samples were not given antibiotics.

Immediately after diagnosis and prior to treatment (Day 1), milk samples (~10–20 mL) were aseptically collected from the affected udder quarters and stored at 4 °C. Aseptic quarter milk samples were also collected over the next two days (days 2 and 3) at approximately 24 h intervals. In addition, aseptic milk samples were collected by farm technicians later in the lactation cycle (March 2023) from the same udder quarter in cows that had recovered from acute mastitis and were still part of the dairy herd.

Acute mastitis quarter milk samples collected from cows on day 1 were subjected to on-farm microbiological testing using Mastatest (MastaPlex, Hamilton, New Zealand) [21], standard laboratory-based microbial tests (see below) and biochemical testing. Milk samples from the same quarter collected on days 2 and 3 were only subjected to laboratory-based microbial tests. Milk assigned for bacteria analysis were kept at 4–8 °C until testing (within 12 h). All milk samples for biochemical analysis were immediately centrifuged at 6000 *g* for 10 min at 10 °C. The milk supernatant was carefully collected to prevent carry-over of milk fat by filtering through gauze before being frozen as fat-free supernatants in aliquots at −20 °C until analysis.

#### 2.1.3. Healthy and Subclinical Mastitis Milk Collection and Analysis

Pooled milk samples from ~1100 cows (peak lactation, late September 2023) were collected from the farm as part of a herd test (LIC, Hamilton, New Zealand). These were numbered, tested for milk somatic cell count (SCC) by LIC (Fossomatic FT120, FOSS A/S, Hillerød, Denmark) and then sent to Koru Biotech (Palmerston North, New Zealand) as fat-free supernatants for analysis. These were kept frozen at −20 °C until biochemical analysis.

### 2.2. Milk Tests

#### 2.2.1. Lactate Dehydrogenase (LDH) Activity

As an indicator of udder inflammation-induced tissue damage [22], milk LDH activity was used as a confirmatory test of clinical moderate to severe mastitis and was measured using a standard kinetic enzyme assay. Briefly, 20 µL milk (diluted to 2.5% or 5% in 30 mM Tris/HCl buffer pH 8.5 containing 1 mM EDTA plus or minus 5 mM oxalic acid) was added to 130 µL reaction solution (Tris-HCl, EDTA buffer containing 4 mM sodium pyruvate, 0.15 mM reduced NADH), and the change in absorbance (λ 340 nm) was monitored over 10 min at 21 °C (SpectroMax plate reader, Molecular Devices, San Jose, CA, USA). Milk LDH activity was calculated using the extinction coefficient for NADH (ε340 = 6220 M^−1^cm^−1^) and absorbance after subtraction of corresponding oxalic acid values over 10 min and expressed as µmoles/min/L. Positive (milk [quarter] from a cow with acute *E. coli* mastitis) and negative (milk [quarter] from a healthy cow) controls were included in each run to ensure assay consistency.

#### 2.2.2. sLPS/IgG ELISA

**Scheme 1:**
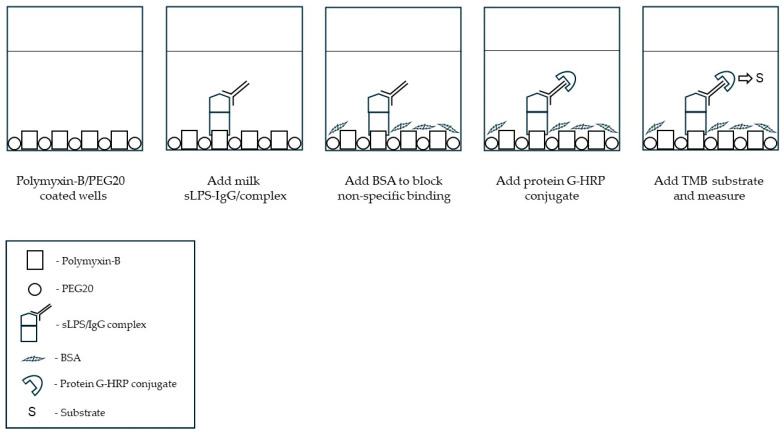
Schematic diagram of sLPS/IgG ELISA format.

The presence of sLPS/IgG in milk was determined using our “in-house” ELISA format involving the capture of LPS (via its conserved lipid A/KDO region) using a polymyxin B capture-based method described previously [20] and shown in a schematic diagram (Scheme 1) below. Milk supernatants were pretreated (1:2 [*v*/*v*] dilution) in 0.1 M sodium citrate, pH 5.4, containing 0.025% Triton X100 for 30 min at RT (21 °C) and immediately measured by the ELISA. Briefly, milk dilutions (100 µL) were added to a polymyxin B-coated plate and incubated for 90 min at 37 °C. After washing (4×) with PBS, pH 7.4, containing 0.1% Tween-20 (PBST), non-specific binding was blocked using 2% BSA/PBS, pH 7.4, for 30 min at RT. The presence of sLPS/IgG complexes were detected using HRP-conjugated protein G (diluted 1:8000 in PBST, purchased from Invitrogen Life Technologies, Invitrogen, Carlsbad, CA, USA; Cat#P21041); HRP-conjugated chicken anti-bovine IgG (diluted 1:3000 in PBST, purchased from Invitrogen Life Technologies; Invitrogen, Carlsbad, CA, USA; Cat#1157540) was used initially to verify the specificity of the protein G conjugate. Incubation for 45 min at 37 °C, after addition of the HRP conjugate (100 µL) to wells, was followed by washing plates with PBST (×4) and detection of peroxidase activity using TMB solution. The reaction was stopped after 15 min using 10% (*v*/*v*) sulphuric acid (50 µL) and absorbance read at 450 nm and 650 nm using a SpectroMax plate reader (Molecular Devices, San Jose, CA, USA). Results were calculated as absorbance 450 nm minus 650 nm (reference value). Positive (milk [quarter] from a cow with acute *E. coli* mastitis) and negative (milk [quarter] from a healthy cow) controls were included to ensure absorbance consistency across plates.

#### 2.2.3. Microbiological Tests

Milk samples (~10 µL) were plated on sheep blood agar and MacConkey agar plates. Plates were incubated at 37 °C and evaluated after 24 and 48 h. Colonies of interest were identified on a species level using Matrix-Assisted Laser Desorption Ionization Time of Flight Mass Spectrometry (MALDI-TOF MS, MALDI Biotyper, Bruker Daltonics, Bremen, Germany) analysis. To aid the identification by MALDI-TOF MS, some bacterial isolates were sub-cultured on sheep blood agar plates. Samples from which a single colony type was cultured followed by species-specific identification of the pathogen by MALDI-TOF MS were assigned to either the Gram-positive or Gram-negative sample group. Samples from which two or more colony types were isolated and identified were assigned to the polymicrobial infection group.

#### 2.2.4. Statistical Analysis

Analysis and graphical representation of the data was performed, except where noted, using Microsoft Excel Analysis ToolPak statistical software (Microsoft 365). Data represented graphically as box-and-whisker plots showed a box spanning the interquartile range (IQR), with the lower and upper edges representing the first (Q1) and third (Q3) quartiles, and the central line indicating the median. Whiskers extend to the most extreme data points within 1.5× the IQR from the quartiles, and values beyond this range are suspected outliers (depicted as circles).

All grouped data variables were assessed for normality using both qualitative (Quantile-Quantile plot) and quantitative (Kolmogorov–Smirnov test) analysis prior to statistical analysis. Comparison of data variables between data groups was assessed for mean differences with 95% CI. The mean plus 3 × SD value (i.e., within a confidence level of 99.7%) of sLPS/IgG absorbance values within the healthy group was used to establish the sLPS/IgG assay threshold. ROC curve (AUC) was used to assess the ability of the sLPS/IgG assay threshold value to distinguish between the three disease categories (acute mastitis, subclinical mastitis and healthy) as well as assay accuracy within 95% CI. In cows with acute mastitis, an ordinal logistic generalised estimating equation was used to examine the effect of bacterial cause (Gram-positive only, Gram-negative only and polymicrobial) on day, infection type and their interaction as predictor variables. This analysis was undertaken using SPSS version 30 (IBM, New York, NY, USA).

## 3. Results

### 3.1. Establishing Healthy and Mastitis Milk Groups

#### 3.1.1. Healthy and Subclinical Mastitis Milk Group Selection

**Figure 1:**
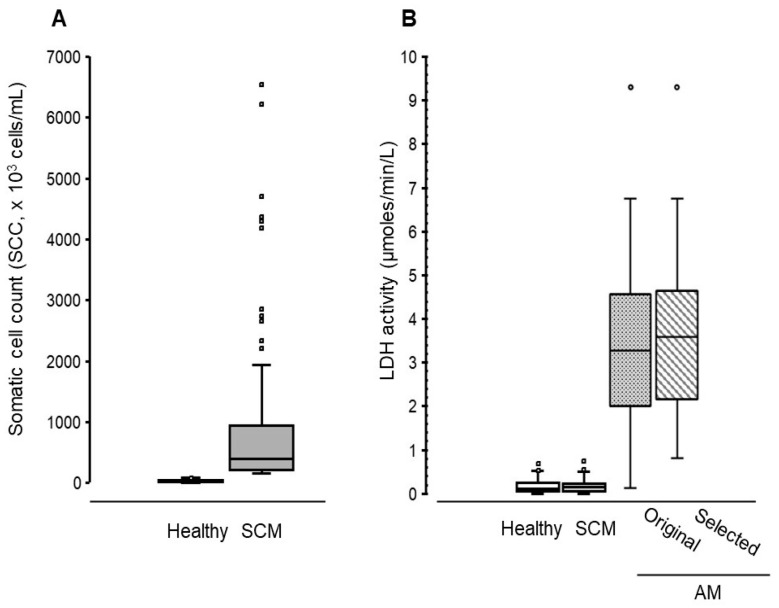
Box-and-whisker plots show (**A**) somatic cell count (SCC, ×10^3^ cells/mL) in pooled quarter milk samples from healthy (*n* = 146) and subclinical mastitis (SCM, *n* = 135) groups, and (**B**) LDH activity in pooled quarter milk samples from the healthy (*n* = 146) and SCM (*n* = 135) groups or quarter milk samples from the acute mastitis group (AM; original (*n* = 34) and selected (*n* = 32). ◦ likely outlier.

Using the SCC herd testing results provided by LIC, we separated the data into two groups: (i) SCC values < 100 × 10^3^ cells/mL (healthy) and (ii) >150 × 10^3^ cells/mL (subclinical mastitis). Using these data groups, 150 samples were sequentially selected from each SSC data group until a total of 300 was achieved (~30% of the total herd at the time). Of these 300 pooled milk samples, 281 had sufficient volume for biochemical analysis. There were 146 samples in the healthy group with a median (IQR) of 22 (13.25 to 41) × 10^3^ cells/mL, whereas the subclinical mastitis group contained 135 samples with a median (IQR) SCC of 395 (208.5 to 949.5) × 10^3^ cells/mL (Figure 1A). Additional animal information is available in the Supplementary Data Section (Supplementary Table S1).

#### 3.1.2. Acute Mastitis Group Representing Moderate–Severe Intramammary Inflammation

Cows age and days in milk (DIM) at the detection of, and recovery from, acute mastitis is shown in Table 1. Milk (quarter) samples from 34 cows showed signs of acute mastitis within 71 days from the start of lactation (median (IQR) 33 (7 to 55).

The milk (quarters) from the 34 cows with clinical signs of acute mastitis showed LDH activity ranging from 0.13 to 9.30 µmoles/min/L (median of 3.28). In contrast, pooled milk samples from the healthy and subclinical mastitis groups showed LDH activity ranging from 0 to 0.69 (median = 0.12) and 0 to 0.82 (median = 0.15) µmoles/min/L, respectively (Figure 1B). Representative data from 20 separate assays showed mean µmoles/min/L (±SD) of 0.001 ± 0.0005 and 7.31 ± 0.81, for negative and positive milk controls, respectively, with an inter-assay variation of 27.5%.

Our aim here was to evaluate milk sLPS/IgG complex levels as potential diagnostic indicators of moderate-to-severe udder inflammation. Since no formal clinical scoring of acute mastitis was applied in this pilot study, we used milk LDH activity (which increases with the severity of clinical mastitis [23]) as a means to preselect milk samples that not only displayed clinical signs but also had an elevation in LDH activity. In a previous study by us [20], we demonstrated that cows with moderate-to-severe *E. coli* mastitis showing milk LDH activity >0.80 µmoles/min/L also showed increases in sLPS/IgG complexes levels. Since the same assay methodology is applied in this study, we chose this LDH value as a threshold for selecting acute mastitis milk samples with moderate-to-severe inflammation. We found here that milk from 32 out of 34 cows showing signs of acute mastitis fulfilled these criteria. The selected 32 acute mastitis milk samples showed a normal distribution of LDH activity ranging from 0.81 to 9.30 µmoles/min/L with a median of 3.63 (Figure 1B).

### 3.2. Association Between Milk sLPS/IgG Complex Levels and Mastitis Status

#### 3.2.1. Milk sLPS/IgG Complex Levels in Healthy and Mastitis Cows

The “in-house” diagnostic sensitivity of the ELISA was determined by comparing sLPS/IgG absorbance values of buffer only (background, *n* = 90 observations) with milk quarter samples collected from five healthy mid-lactation cows from four separate commercial dairy herds (*n* = 80). Mean background absorbance was 0.004 ± 0.003, showing an assay limit of detection (mean + 3 × SD) of 0.0145. Healthy milk absorbance values ranged from 0.013 to 0.30 (median [IQR] 0.05 [0.03 to 0.09]). Mean difference from background absorbance was 0.06 (95% CI, 0.0004 to 0.12). Specificity of the “in-house” sLPS/IgG ELISA has been examined in a previous study [20]. Furthermore, representative data from 11 ELISA plates showed mean absorbance (± SD) of 0.015 ± 0.002 and 1.22 ± 0.104, for negative and positive controls, respectively, with an inter-assay variation of 12%.

**Figure 2:**
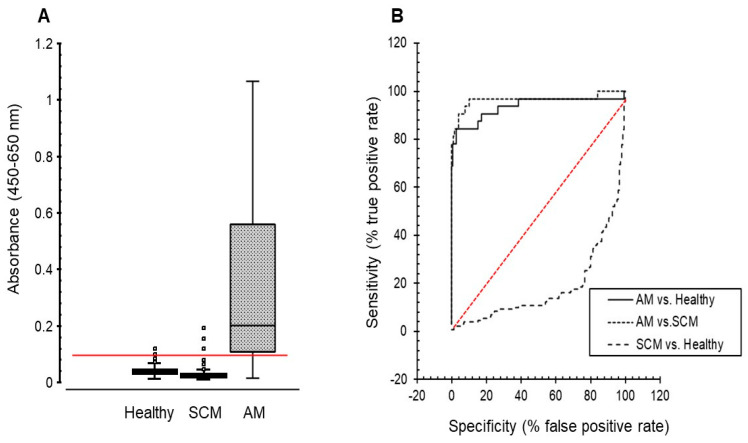
(**A**) represents a box and whisker plot of sLPS/IgG absorbance values in pooled quarter milk samples from healthy cows (*n* = 146) and those with subclinical mastitis (SCM, *n* = 135) or quarter milk samples from the acute mastitis group (AM, *n* = 32). The solid red line represents an assay absorbance threshold of 0.089. (**B**) represents a receiver operating characteristic (ROC) curve showing a differentiation in AM (active) variables from those in both healthy (solid black line) and SCM (dashed black line) groups. The wide-dashed black line signifies the differentiation between healthy and SCM sLPS/IgG values and the red dashed line a random differentiation. ◦ likely outlier.

In this pilot study, milk sLPS/IgG absorbance values were normally distributed in all three groups, and ranged from 0.01 to 0.12 in healthy milk from 0.01 to 0.19 in the subclinical mastitis group, and from 0.02 to 1.68 in the acute mastitis group (Figure 2A). Mean milk sLPS/IgG absorbance in the acute mastitis group was 0.39 (95% CI, 0.23 to 0.55), while mean absorbance in the healthy and subclinical mastitis groups were 0.04 (95% CI, 0.037 to 0.043) and 0.03 (95% CI, 0.027 to 0.033), respectively. Cows with acute mastitis had markedly greater sLPS/IgG absorbance values than healthy cows (mean difference 0.35; 95% CI, 0.28 to 0.42) and cows with subclinical mastitis (0.36; 95% CI, 0.28 to 0.44), respectively. In contrast, the mean difference in sLPS/IgG absorbance values between healthy cows and those with subclinical mastitis was small (0.01; 95% CI, 0.005 to 0.015) and unlikely to be biologically relevant.

Threshold of the sLPS/IgG assay (0.089) was calculated using mean ± 3 × SD of absorbance values detected in healthy milk samples. Application of this threshold value in ROC analysis (Figure 2B) revealed that the milk sLPS/IgG assay could distinguish cows with acute mastitis from healthy ones with 100% specificity and 78% sensitivity. Calculation of the area under the ROC curve (AUC) also showed the accuracy of the sLPS/IgG assay to be high (AUC = 0.94 [95% CI, 0.9 to 0.97]). A similar assay performance (AUC = 0.97 [95% CI, 0.9 to 0.97]) for acute versus subclinical mastitis using the same threshold value (0.089 absorbance) was also found, where cows with acute mastitis were distinguished from those with subclinical mastitis with a specificity of 99% and a sensitivity of 78%. However, application of this assay threshold value (0.089 absorbance) failed to distinguish cows with subclinical mastitis from healthy cows with ROC analysis showing an assay sensitivity of only 1.5%, whilst retaining a specificity for LPS/IgG detection of 100% and a high assay accuracy (AUC = 0.94 [95% CI, 0.94 to 0.99).

#### 3.2.2. Comparison of Milk sLPS/IgG Levels and LDH Activity at the Detection of Acute Mastitis and After a Period of Recovery

A total of 20 out of the 32 cows identified with acute mastitis in early lactation (DIM; median [IQR]: 33 [6.8 to 55]) were present in the herd later in the lactation cycle (DIM; 199.5 [185.25 to 209.75]); the reminder had been sold, culled or died. In the latter stage of lactation, these cows showed no apparent signs of clinical mastitis and had milk SCC (pooled quarter samples) values ranging from 22 to 3192 (median [IQR] 119.5 [44.28 to 472.75]) × 10^3^ cells/mL.

**Figure 3:**
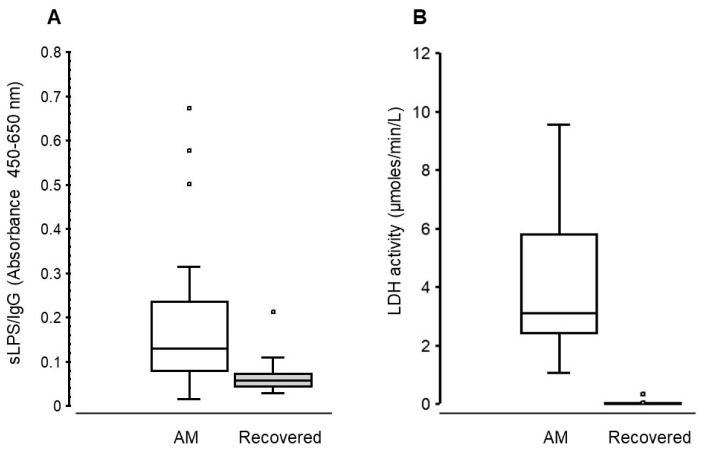
Box-and-whisker plots displaying (**A**) sLPS/IgG levels and (**B**) LDH activity in paired quarter milk samples from cows (*n* = 20) at the detection of acute mastitis (AM) and then later in the lactation cycle (recovered). ◦ likely outlier.

We assessed sLPS/IgG levels (Figure 3A) and LDH activity (Figure 3B) in the same udder quarters that were previously affected by mastitis. Milk sLPS/IgG values ranged from 0.02 to 0.67 (median [IQR] 0.13 [0.08 to 0.24], whereas milk LDH activity ranged from 1.06 to 9.56 (median [IQR] 3.11 [2.43 to 5.78] µmoles/min/L). In recovery milk samples sLPS/IgG absorbance values ranged from 0.03 to 0.21 (median [IQR] 0.06 [ 0.04 to 0.07]), whereas LDH activity ranged from below the threshold to 0.34, respectively. The mean difference (mastitis vs. recovery) in sLPS/IgG absorbance between these paired quarter milk samples was 0.13 (95% CI; −0.01 to 0.27). Similarly, the mean difference in milk LDH activity was 4.47 (95% CI: 0.01 to 8.91).

### 3.3. Milk sLPS/IgG Levels and Bacterial Causes of Acute Mastitis

#### 3.3.1. Bacteria Strains Detected in Acute Mastitis Milk

Bacteria were cultured from all 32 acute mastitis samples which had LDH > 0.8 µmoles/min/L. The bacterial species identified using MALDI-TOF are summarised in Table 2. This enabled the separation of acute mastitis milk into three bacteria groups: (i) Gram-positive (single) infection [*n* = 6], (ii) Gram-negative (single) infection [*n* = 5] or (iii) polymicrobial infection (shown to contain both Gram-positive and negative bacteria) [*n* = 21].

#### 3.3.2. Milk sLPS/IgG Association with Bacterial Acute Mastitis

**Figure 4:**
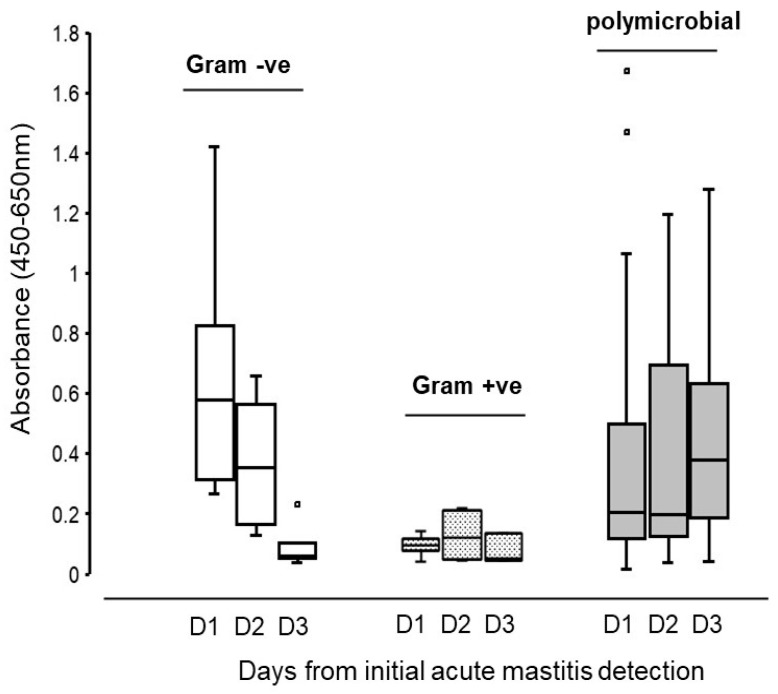
Box-and-whisker plots of sLPS/IgG absorbance values in acute mastitis Gram-negative (gram -ve), Gram-positive (gram + ve) and polymicrobial bacterial quarter milk groups, over the 3 days from initial detection: day 1 (D1), day 2 (D2) and day 3 (D3), respectively. ◦ likely outlier.

Milk sLPS/IgG levels displayed a differential pattern within each of the three milk bacteria groups over the first 3 days (day 1 to day 3) from the initial detection of acute mastitis (Figure 4). The Gram-negative bacteria-only group showed sLPS/IgG levels ranging from 0.27 to 1.42 (median = 0.58) on day 1 (i.e., day of acute mastitis detection), and from 0.13 to 0.66 (median = 0.35) and from 0.04 to 0.23 (median = 0.06) on days 2 and 3, respectively. In contrast, sLPS/IgG absorbance in acute mastitis milk containing only Gram-positive bacteria ranged from 0.04 to 0.14 (median = 0.1), from 0.05 to 0.22 (median = 0.12) and from 0.04 to 0.14 (median = 0.05) on days 1, 2 and 3, respectively. In acute mastitis milk samples that were polymicrobial, sLPS/IgG absorbance values ranged from 0.02 to 1.67 (median = 0.21), from 0.04 to 1.20 (median = 0.20) and from 0.04 to 1.28 (median = 0.38) on days 1, 2 and 3, respectively.

Compared to sLPS/IgG absorbance values detected on day 1 within the polymicrobial group, the odds of sLPS/IgG absorbance values being higher on day 2 than day 1 was 1.2 (95% CI, 0.55 to 2.6), while for day 3 versus day 1 the equivalent odds were 1.83 times higher (95% CI, 0.62 to 5.3). So, while our results were compatible with no change in milk sLPS/IgG levels over time in samples from cows with polymicrobial infections, they were also compatible with a wide range of effects from a moderate decrease in odds (lower confidence limits for day 2 versus day 1 and day 3 versus day 2 were 0.55 and 0.62, respectively) to a large increase (upper confidence limits for day 2 versus day 1 and day 3 versus day 2 were 2.6 and 5.3, respectively). The odds of a day 1 milk sample from which a single species of Gram-negative bacteria had been isolated having a higher sLPS/IgG absorbance than a day 1 sample from the polymicrobial group were 4.4 times higher (95% CI, 0.95 to 20.2). Thus, although our preliminary data were compatible with a large increase in odds, they were also compatible with no biologically important difference. In contrast, our data supported the conclusion that day 1 milk sample from which a single species of Gram-positive bacteria had been isolated were likely to have a lower sLPS/IgG absorbance than a day 1 sample from the polymicrobial group (odds ratio (OR) 0.20 times higher (95% CI, 0.03 to 0.149).

However, the interaction between day and infection meant that the relationship between sLPS/IgG levels and infection type depended upon the day. For sLPS/IgG absorbance values in milk from which a single species of Gram-negative bacteria had been isolated, the analysis suggests that compared to the changes between day 1 and day 2 for polymicrobial samples, sLPS/IgG levels had lower odds of being higher on day 2 than day 1 for milk containing Gram-negative (single) bacteria (OR 0.25 [95% CI 0.0.04 to 1.5]), but that our data were compatible with a range of effects from a large decrease in odds to a large increase. In contrast, for days 1 to 3 comparisons, there was a clear large reduction in odds compared to polymicrobial samples (OR 0.20 [95% CI, 0.003 to 0.15]), i.e., samples from which a single species of Gram-negative bacteria had been isolated were much more likely to have a lower sLPS/IgG absorbance on day 3 compared to day 1 than polymicrobial samples. For milk samples from which a single species of Gram-positive bacteria had been isolated, the analysis was inconclusive. For day 1 to day 2 comparison with polymicrobial samples, our results were compatible with both a large decrease in odds of sLPS/IgG levels being higher than that and a large increase (OR 1.03 [95% CI, 0.25 to 4.3]). For day 1 to day 3 comparison, our data were compatible with both a large decrease in odds and a moderate increase (OR 0.27 [95% CI, 0.06 to 1.22]).

## 4. Discussion

Cows early in lactation are exposed to various stress factors that together compromise immune defences. Therefore, postpartum cows are more susceptible to opportunistic environmental bacterial infections that can cause acute mastitis of various severities [1,24]. In this pilot observational study, the primary source of Gram-negative bacterial acute mastitis was environmental and appeared to have originated from a farm feed pad, which was compounded by the wet weather experienced during spring calving season in the southern regions of the North Island. Gram-negative bacteria generate specific LPS compounds that can be endotoxic and drives the acute inflammatory response observed in Gram-negative infections [7,25]. The interaction of IgG with specific bacterial products provides the formation of immune complexes and an insight into the local pro- and anti- inflammatory dynamics of these intramammary IgG complexes within the udder during a bacterial infection. In a previous study [20], we developed a LPS ELISA that was capable of specifically detecting LPS compounds from a range of Gram-negative bacteria associated with mastitis in dairy cows [9,12,26]. Modification of this ELISA format enabled the capture and detection of sLPS/IgG complexes in milk; however, the lack of a sLPS/IgG complex standard (currently in development) does limit the analytical specificity and sensitivity interpretation of our findings. Nevertheless, results showing significant milk sLPS/IgG absorbance differences between healthy cows from those with acute mastitis do support a diagnostic potential of this ELISA format to detect pre-formed sLPS/IgG complex within affected udder quarters during mastitis. Therefore, in this pilot study, we provide evidence that increased milk-soluble (s) LPS/IgG complex levels at the time of acute mastitis detection in dairy cows support a link between mastitis severity and intramammary Gram-negative infections during early lactation.

Recruitment of antigen-specific IgG during a bacterial infection leads to the formation of antigen/IgG complexes that elicits an immune response. As part of this process, the functional properties of these complexes can be diverse, exhibiting concomitant inflammatory and protective properties, which are dependent upon the underlying cause. Here, despite continuous exposure of the mammary gland to environmental Gram-negative bacteria (and their endotoxins), the milk from healthy cows during peak lactation showed low levels of sLPS/IgG levels. These findings may be the result of low intramammary IgG levels identified in healthy cows [2] and thus the unavailability of IgG to form LPS/IgG complexes. In contrast, the rapid early selective transfer of antigen-specific IgG antibodies from blood into the udder during a bacterial infection correlates with the severity of intramammary inflammation [27]. Hence the formation and presence of soluble LPS/IgG complexes in milk is most likely associated with parallel recruitment of specific anti-LPS IgG into the mammary gland during Gram-negative infections. Here, however, we analysed milk collected using different sampling strategies, which limits our interpretation of these findings. Whilst the use of quarter milk samples from acute mastitis and cows that had recovered from acute mastitis support differences in sLPS/IgG complex levels in healthy and mastitis, the use of only pooled milk samples collected from healthy or cows with subclinical mastitis may mask a low elevation of sLPS/IgG levels in an individual quarter. Therefore, further studies are required to verify sLPS/IgG complex differences in milk from subclinical and clinical mastitis cases on a quarter level.

Intramammary infections with Gram-negative bacteria tend to result in a wide range of mastitis severities [6,7]. Since mastitis causes an increase in both milk LDH activity and total IgG, it is possible that increases in milk LDH activity may also be associated with increases in the intramammary formation of sLPS/IgG complexes during Gram-negative bacteria mastitis. The observation of a reduction in both sLPS/IgG levels and LDH activity in udder quarters previously affected by mastitis suggests that sLPS/IgG levels may reflect inflammation involving Gram-negative pathogens. Our previous findings of a link between milk sLPS/IgG complex levels and mastitis severity caused by *E. coli* [20] are supportive of this finding. However, neither endotoxicity nor the induction of LPS-related pro-inflammatory mediators, as reported by others [11,12,20], were measured in this pilot study. Advances in diagnostic technologies, such as gene microbial mapping, show acute mastitis cases in early lactating dairy cattle tend to be polymicrobial [28]. Since the aetiology of mastitis can involve other microbes (including fungi, algae), we cannot exclude the possibility that other unknown pathogens may have contributed to disease severity and prognosis, and even though this is a pilot study, we found 60% of acute mastitis milk samples were classified as polymicrobial (i.e., shown to contain both Gram-negative and Gram-positive bacteria). Increases in milk sLPS/IgG levels at the detection of acute mastitis was identified in samples yielding single Gram-negative bacteria and polymicrobial cultures, but not when only Gram-positive bacteria were identified. This supports the link between milk sLPS/IgG levels and mastitis caused by Gram-negative bacteria. These current findings are limited, however, to a relatively small sample set and may have been affected by inherent weaknesses of microbiological techniques employed [5]. In addition, the temporal dynamics of milk sLPS/IgG may be influenced by other factors, such as environmental changes (interbacterial combined virulency and/or efficacy [29,30]) and mastitis treatment strategies [30]. Our preliminary findings, even with a limited number of mastitis milk samples, indicate that treatment approaches may influence disease dynamics. Assessment of sLPS/IgG levels may offer insight in treatment impacts on the progression and duration of disease involving Gram-negative infections. This, however, requires further study.

## 5. Conclusions

The findings from this pilot study support milk sLPS/IgG complexes as a link between mastitis severity and intramammary Gram-negative infections. This is especially informative since it can be difficult to identify the relevance of Gram-negative bacteria in polymicrobial infections or contaminated samples using solely microbiological testing procedures. These conclusions, however, have limitations, including the use of a single herd within a short period (8 weeks) during early lactation and using milk LDH activity > 0.8 µmoles/min/L as a means of selecting cows displaying signs of moderate mastitis. In addition, since the prevalence of acute mastitis within this herd is ~5% (average incidence in NZ can be up to 15% [31]), the number of cows recruited into this pilot study was low (*n* = 34). Further studies are required to support the efficacy of milk sLPS/IgG complexes to differentiate between subclinical and clinical mastitis Gram-negative infections as well as a potential indicator to identify the impact mastitis treatment has on dairy herd management.

## 6. Patents

Concepts described in this manuscript are part of a patent submission by Koru Diagnostics Ltd., entitled “Method for Detecting Microorganisms and Uses Thereof” in November 2022. Patent Ref: PCT/NC2022/050136, #KDL1007PC.

## Figures and Tables

**Table 1:** Animal information: Acute mastitis group (*n* = 34).

**Age at Diagnosis (yrs)**	
Median (IQR) Range	6 (3.25 to 8) 2 to 10
**Days in milk**	
Detection of acute mastitis (*n* = 34)	
Median (IQR) Range	33 (7 to 55) 1–71
**Days in milk**	
Recovered (*n* = 20)	
Median (IQR) Range	200 (185 to 210) 161–224

**Table 2:** Summary of bacteria identified in milk cultures from cows with acute mastitis (*n* = 32).

Gram-Positive (Single Species) (*n* = 6)	Gram-Negative (Single Species) (*n* = 5)	Polymicrobial (*n* = 21)
*Streptococcus uberis* (*n* = 4)	*Escherichia coli* (*n* = 3)	*Streptococcus uberis* + *Escherichia coli* (*n* = 10)
*Streptococcus dysgalactiae* (*n* = 1)	coliforms (*n* = 2)	*Staphylococcus aureus* + *Escherichia coli* (*n* = 4)
*Staphylococcus aureus* (*n* = 1)		*Streptococcus* spp. + coliforms (*n* = 6)
		*Streptococcus* spp., *Staphylococcus* spp., coliforms (*n* = 1)

## Data Availability

Raw data generated during this study are not readily available as they are proprietary and part of a pending patent submission. However, requests to access specific data should be directed to Koru Biotech Solutions Ltd. info@korubiotech.com.

## Supplementary Materials

The following supporting information can be downloaded at https://www.mdpi.com/article/10.3390/ani16020310/s1, Supplementary Table S1: Age and Days in milk (DIM) for Healthy and Sub-clinical mastitis groups.

## References

[1] Meçaj R., Muça G., Koleci X., Sulçe M., Turmaiaj L., Zalla P., Koni A., Tafaj M. (2023). Bovine environmental mastitis and their control: An overview. Int. J. Agric. Biosci..

[2] Viasova A.N., Saif L.J. (2021). Bovine immunology: Implications for dairy cattle. Front. Immunol..

[3] Stanek P., Żólkiewski P., Januś E. (2024). A review on mastitis in dairy cows research: Current status and future perspectives. Agriculture.

[4] Rasmussen M.D., Bjerring M., Skjøth F. (2005). Visual appearance and CMT score of foremilk of individual quarters in relation to cell count of cows milked automatically. J. Dairy Res..

[5] Rowe S., House J.K., Pooley H., Bullen S., Humphris M., Ingenhoff L., Norris J.M., Zadoks R. (2023). Evaluation of point-of-care tests for identification of pathogens to inform clinical mastitis treatment decisions in pasture-and confinement-managed dairy cows in Australia. J. Dairy Sci..

[6] Fredebeul-Krein F., Schmenger A., Wente N., Zhang Y., Krömker V. (2022). Factors associated with the severity of clinical mastitis. Pathogens.

[7] Krebs I., Zhang Y., Wente N., Leimbach S., Krömker V. (2023). Severity of clinical mastitis and bacterial shedding. Pathogens.

[8] Rasmussen P., Barkema H.W., Osei P.P., Taylor J., Shaw A.P., Conrady B., Chaters G., Muñoz V., Hall D.C., Apenteng O.O. (2024). Global losses due to dairy cattle diseases: A comorbidity-adjusted economic analysis. J. Dairy Sci..

[9] Erridge C., Bennett-Guerrero E., Poxton I.R. (2002). Structure and function of lipopolysaccharides. Micro. Infect..

[10] Jermann P.M., Wagner L.A., Fritsche D., Gross J.J., Wellnitz O., Bruckmaier R.M. (2023). Acute phase reaction to lipopolysaccharide-induced mastitis in early lactation dairy cows fed nitrogenic, glucogenic, or lipogenic diets. J. Dairy Sci..

[11] Johnzon C.-F., Dahlberg J., Gustafson A.-M., Waern I., Moazzami A.A., Östensson K., Pejler G. (2018). The effect of lipopolysaccharide-induced experimental Bovine mastitis on clinical parameters, inflammatory markers, and the metabolome: A kinetic approach. Front. Immunol..

[12] Asfidoajani F.A., Sichani M.M. (2018). Comparison of coliform contamination and endotoxin levels in raw cow’s milk. Zahedan J. Res. Med. Sci..

[13] Flórez P., de Castro M., Rodríguez D., Gonzalo-Orden J.M., Carvajal A. (2023). Intralaboratory validation of a kinetic turbidimetric assay based on *Limulus Amebocyte* lysate (LAL) for assessing endotoxin activity in Cow milk. Animals.

[14] Shahriar F., Gordon J.R., Simko E. (2006). Identification of lipopolysaccharide-binding proteins in porcine milk. Can. J. Vet. Res..

[15] Hinz K., Larsen L.B., Wellnitz O., Bruckmaier R.M., Kelly L.A. (2012). Proteolytic and proteomic changes in milk at quarter level following infusion with Escherichia coli lipopolysaccharide. J. Dairy Sci..

[16] Virzi G.M., Mattiotti M., de Cal M., Ronco C., Zanelle M., De Rosa S. (2023). Endotoxin in sepsis: Methods for LPS detection and the use of omics techniques. Diagnostics.

[17] Rainard P., Gilbert F.B., Germon P. (2022). Immune defenses of the mammary gland epithelium of dairy ruminants. Front. Immunol..

[18] Aibara N., Ohyama K. (2020). Revisiting immune complexes: Key to understanding immune-related diseases. Adv. Clin. Chem..

[19] Chen H., Maul-Pavicic A., Holzer M., Huber M., Salzer U., Chevalier N., Voll R.E., Hengel H., Kolb P. (2022). Detection and functional resolution of soluble immune complexes by an FcγR reporter cell panel. EMBO Mol. Med..

[20] Hurst S.H., Flossdorf D.A.L., Koralagamage R., Pernthaner A. (2024). Selective IgG binding to the LPS glycolipid core found in bovine colostrum, or milk, during *Escherichia coli* mastitis influences endotoxin function. Innate Immunol..

[21] Saila S., Bork O., Tucker I.G., Cranefield S., Bryan M.A. (2023). Evaluation of an on-farm culture system for the detection of subclinical mastitis pathogens in dairy cattle. JDS Commun..

[22] Larsen T. (2005). Determination of lactate dehydrogenase (LDH) activity in milk by a fluorometric assay. J. Dairy Res..

[23] Chagunda M.G.G., Larsen T., Bjerring M., Ingvartsen K.L. (2006). L-lactate dehydrogenase and N-acetyl-β-D-glucosaminidase activities in bovine milk as indicators of non-specific mastitis. J. Dairy Res..

[24] Bates A., Laven R., Bork O., Hay M., McDowell J., Saldias B. (2020). Selective and deferred treatment of clinical mastitis in seven New Zealand dairy herds. Prev. Vet. Med..

[25] Fux A.C., Melo C.C., Michelini S., Swartzwelter B.J., Neusch A., Italani P., Himly M. (2023). Heterogeneity of lipopolysaccharide as source of variability in bioassays and LPS-binding proteins as remedy. Int. J. Mol. Sci..

[26] Moreillon P., Majcherczyk P.A. (2003). Proinflammatory activity of cell-wall constituents from gram-positive bacteria. Scand. J. Infect. Dis..

[27] Khatun M., Thomson P.C., García S.C., Bruckmaier R.M. (2022). Suitability of milk lactate dehydrogenase and serum albumin for pathogen-specific mastitis detection in automatic milking systems. J. Dairy Sci..

[28] Angelopoulou A., Holohan R., Rea M.C., Warda A.K., Hill C., Ross R.P. (2019). Bovine mastitis is a polymicrobial disease requiring a polydiagnostic approach. Int. Dairy J..

[29] Isaac P., Breser M.L., De Lillo M.F., Bohl L.P., Calvinho L.F., Porporatto C. (2025). Understanding the bovine mastitis co-infections: Coexistence with *Enterobacter* alters *S. aureus* antibiotic susceptibility and virulence phenotype. Res. Vet. Sci..

[30] Duse A., Persson-Waller K., Pedersen K. (2021). Microbial aetiology, antibiotic susceptibility and pathogen-specific risk factors for udder pathogens from clinical mastitis in dairy cows. Animals.

[31] Petrovski K.R., Heuer C., Parkinson T.J., Williamson N.B. (2009). The incidence and aetiology of clinical bovine mastitis on 14 farms in Northland, New Zealand. N. Z. Vet. J..

