# Supplementary material for: Pilot Study: Soluble LPS/IgG Milk Complexes in Relationship to Early Lactation Acute Mastitis in Dairy Cows
Source: Animals (Basel). 2026 Jan 20;16(2):310. doi: 10.3390/ani16020310 (PMC12837993; doi:10.3390/ani16020310)
Supplement: Supplementary file 1 [file animals-16-00310-s001.zip › animals-4075136-supplementary.pdf]

Supplementary Table S1: Age and Days in milk (DIM) for Healthy and Sub-clinical mastitis groups

	Healthy	Sub-clinical mastitis	*Mean difference (95% CI)*
Age (yrs)			
• Median (IQR)	4 (3 to 6)	6 (4 to 8)	*1 (1 to 2)*
• Range	2 to 10	2 to 11	
Days in milk (DIM)			
• Median (IQR)	57 (44 to 62)	47 (17 to 59)	*13 (8 to 18)*
• Range	8 to 87	1 to 79	
